# Supplementary material for: Content-rich biological network constructed by mining PubMed abstracts
Source: BMC Bioinformatics. 2004 Oct 8;5:147. doi: 10.1186/1471-2105-5-147 (PMC528731; doi:10.1186/1471-2105-5-147)
Supplement: Additional File 2 — The original results of the above study (non-essential files are deleted to keep the file size under the limit set by BMC bioinformatics). [file 1471-2105-5-147-S2.bz2 › chilibotAdditionalFile2/dip05/91ID9367879E396/html/CBL_KRT18.html]

 


 **CBL** and **KRT18** 
  
Found 1 abstracts in PubMed, retrieved 1.  
 

 What does Google say? 
 PDF only 
| .edu only 

---

**Interactive relationship** (e.g. stimulation, inhibition, etc)

**Neutral relationship**- The screen identified interactions involving c  **Cbl**  and two 14 3 3 isoforms, cytokeratin 18  [ **KRT18** ] , human unconventional myosin IC, and a recently identified SH3 domain containing protein, SH3 P17.  Ref: 9367879 Biochem Biophys Res Commun, 1997
